# Supplementary material for: Mitotic deacetylase complex (MiDAC) recognizes the HIV-1 core promoter to control activated viral gene expression
Source: PLoS Pathog. 2024 May 23;20(5):e1011821. doi: 10.1371/journal.ppat.1011821 (PMC11115230; doi:10.1371/journal.ppat.1011821)
Supplement: S2 Fig — LC-MS/MS analysis was performed on digested proteins from gel spots and identified from acquired MS/MS spectra. Two MS/MS spectra from each protein are shown and annotated with the peptide fragmentation information for sp|Q9H147|DNTTIP1_HUMAN (A,B), sp|Q9H0A0|NAT10_HUMAN (C,D) and sp|Q6PJG2|MIDEAS_HUMAN (E,F). (PPTX) [file ppat.1011821.s003.pptx]

## Slide 1
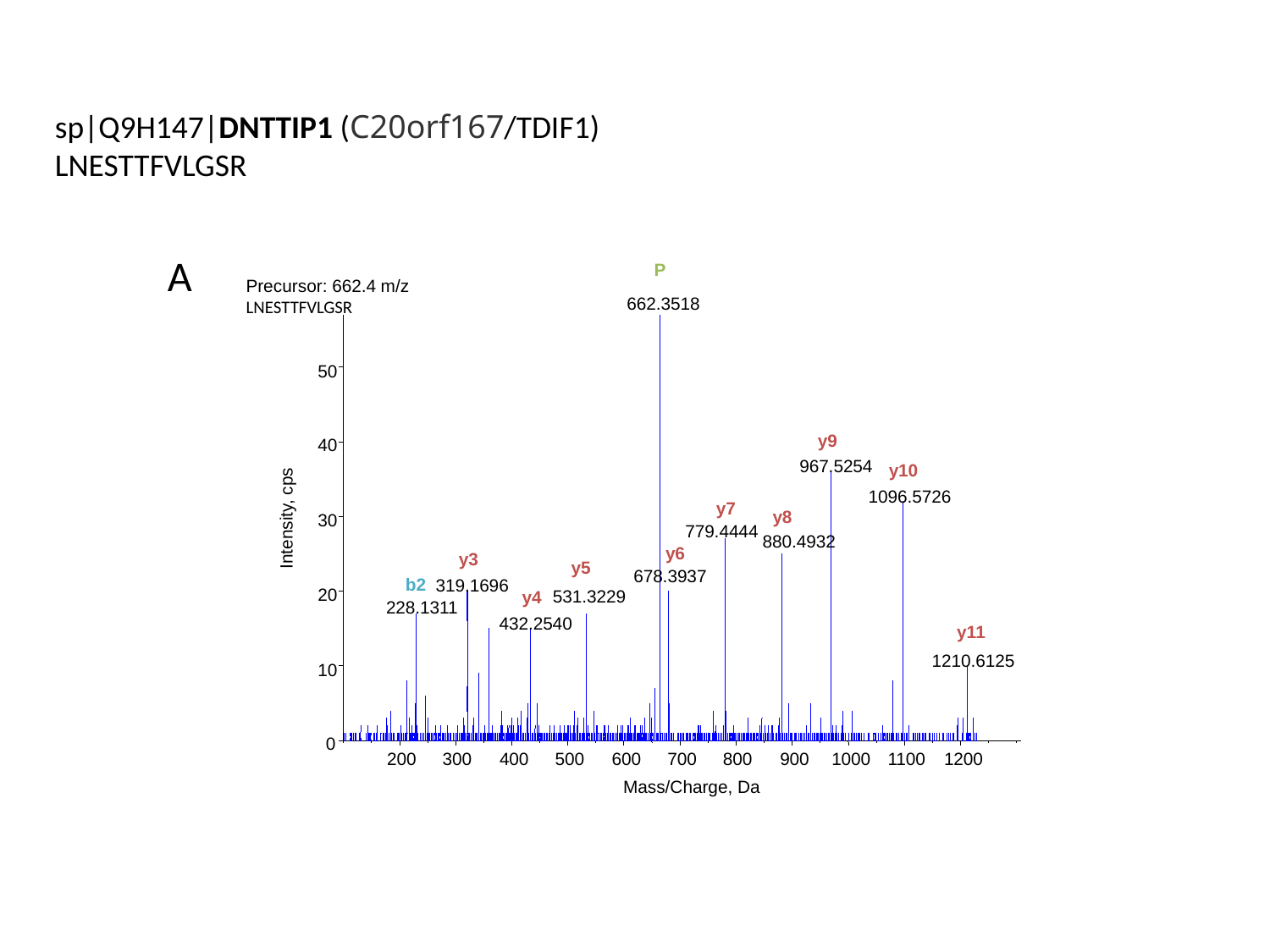

sp|Q9H147|DNTTIP1 (C20orf167/TDIF1)
LNESTTFVLGSR
A
P
Precursor: 662.4 m/z
LNESTTFVLGSR
662.3518
50
y9
40
y10
967.5254
1096.5726
y7
y8
Intensity, cps
30
779.4444
880.4932
y6
y3
y5
678.3937
b2
319.1696
y4
20
531.3229
228.1311
432.2540
y11
1210.6125
10
0
200
300
400
500
600
700
800
900
1000
1100
1200
Mass/Charge, Da

## Slide 2
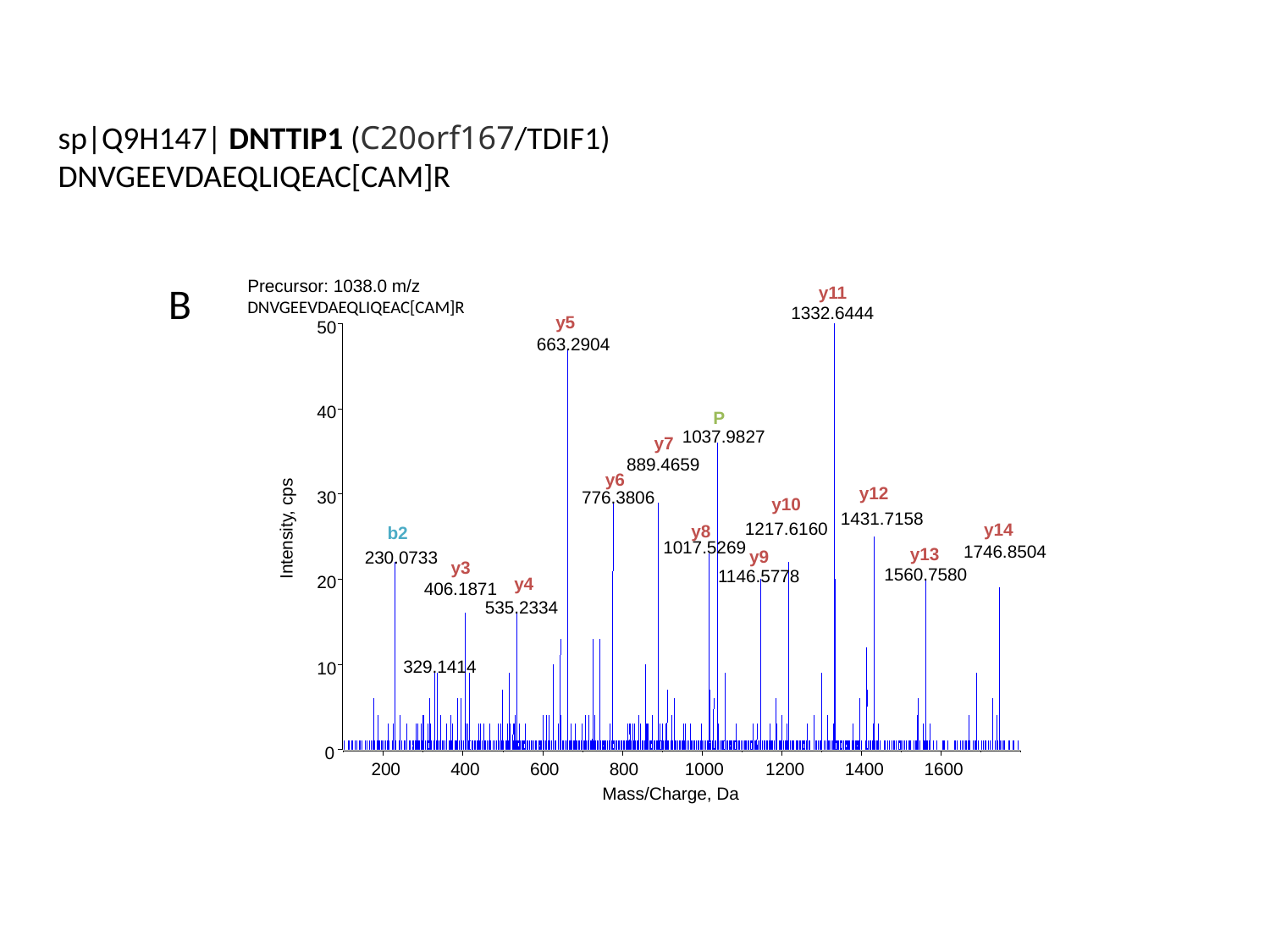

sp|Q9H147| DNTTIP1 (C20orf167/TDIF1)
DNVGEEVDAEQLIQEAC[CAM]R
B
Precursor: 1038.0 m/z
DNVGEEVDAEQLIQEAC[CAM]R
y11
1332.6444
y5
50
663.2904
P
40
y7
1037.9827
889.4659
y6
y12
30
776.3806
y10
1431.7158
y14
y8
b2
Intensity, cps
1217.6160
1017.5269
y13
y9
1746.8504
230.0733
y3
1560.7580
1146.5778
y4
20
406.1871
535.2334
329.1414
10
0
200
400
600
800
1000
1200
1400
1600
Mass/Charge, Da

## Slide 3
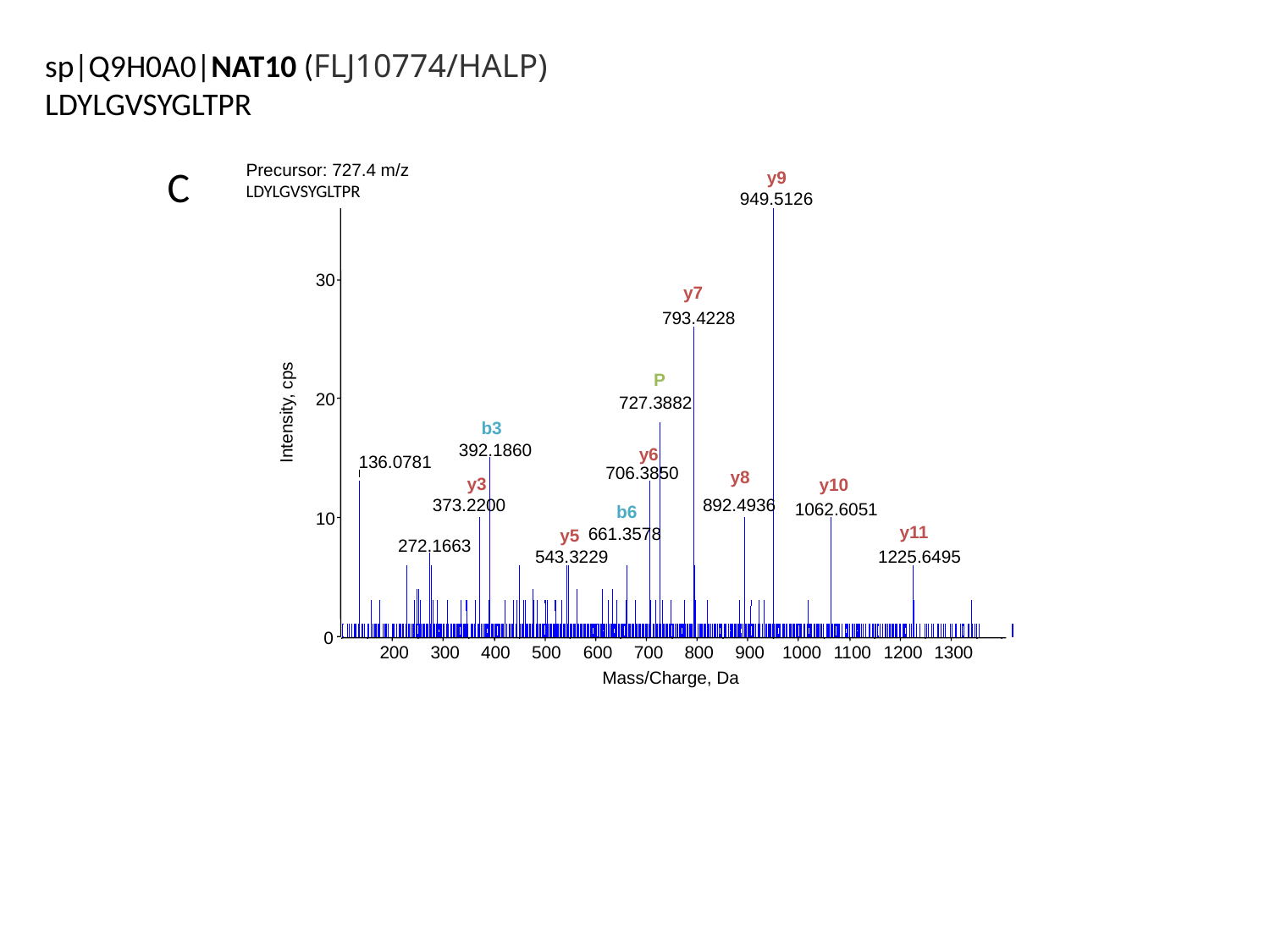

sp|Q9H0A0|NAT10 (FLJ10774/HALP)
LDYLGVSYGLTPR
C
Precursor: 727.4 m/z
LDYLGVSYGLTPR
y9
949.5126
30
y7
793.4228
P
20
727.3882
Intensity, cps
b3
y6
392.1860
136.0781
y8
706.3850
y3
y10
373.2200
892.4936
b6
1062.6051
10
y11
y5
661.3578
272.1663
543.3229
1225.6495
0
200
300
400
500
600
700
800
900
1000
1100
1200
1300
Mass/Charge, Da

## Slide 4
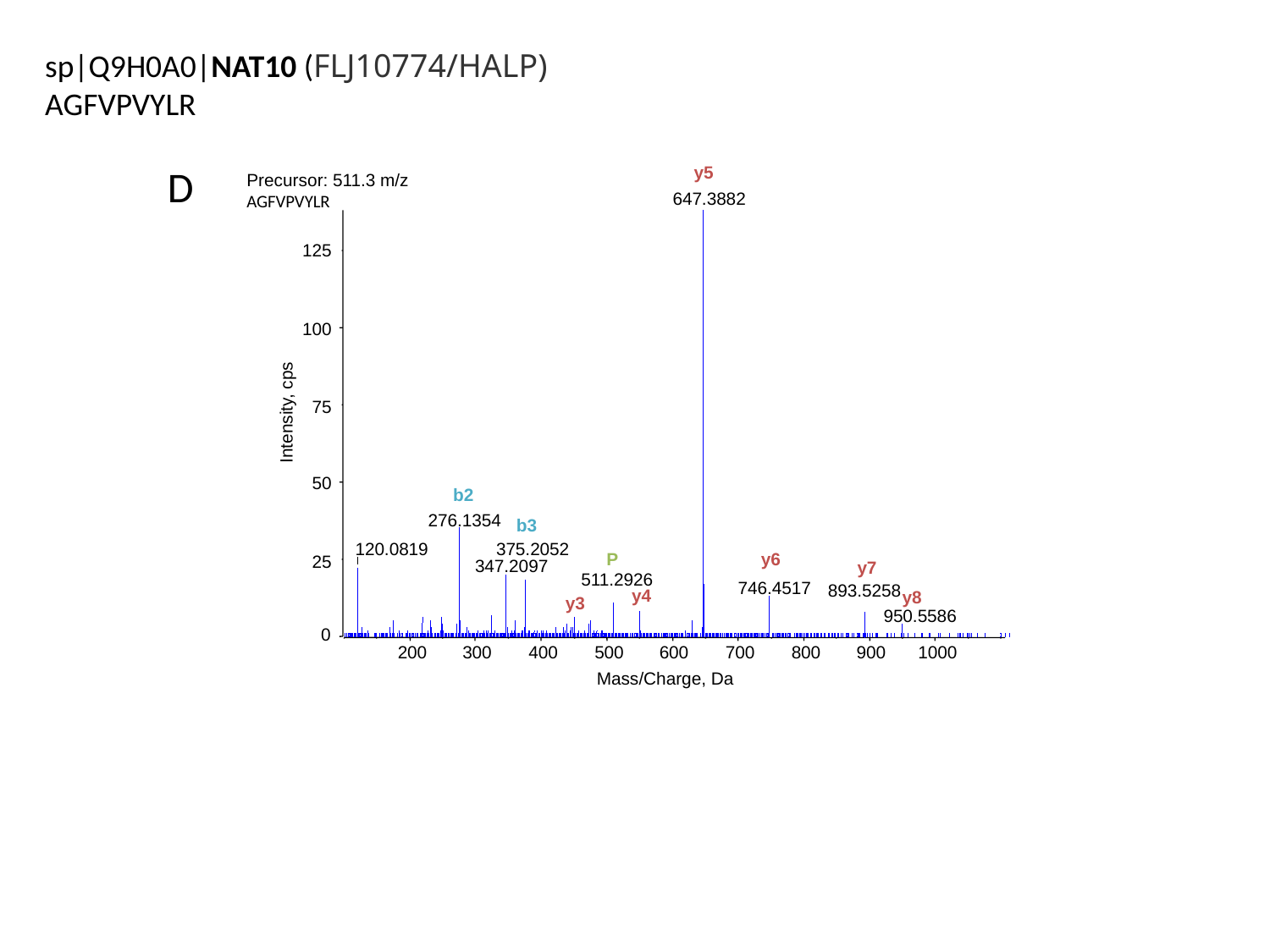

sp|Q9H0A0|NAT10 (FLJ10774/HALP)
AGFVPVYLR
D
y5
Precursor: 511.3 m/z
AGFVPVYLR
647.3882
125
100
75
Intensity, cps
50
b2
b3
276.1354
375.2052
120.0819
P
y6
25
y7
347.2097
511.2926
746.4517
y4
893.5258
y8
y3
950.5586
0
200
300
400
500
600
700
800
900
1000
Mass/Charge, Da

## Slide 5
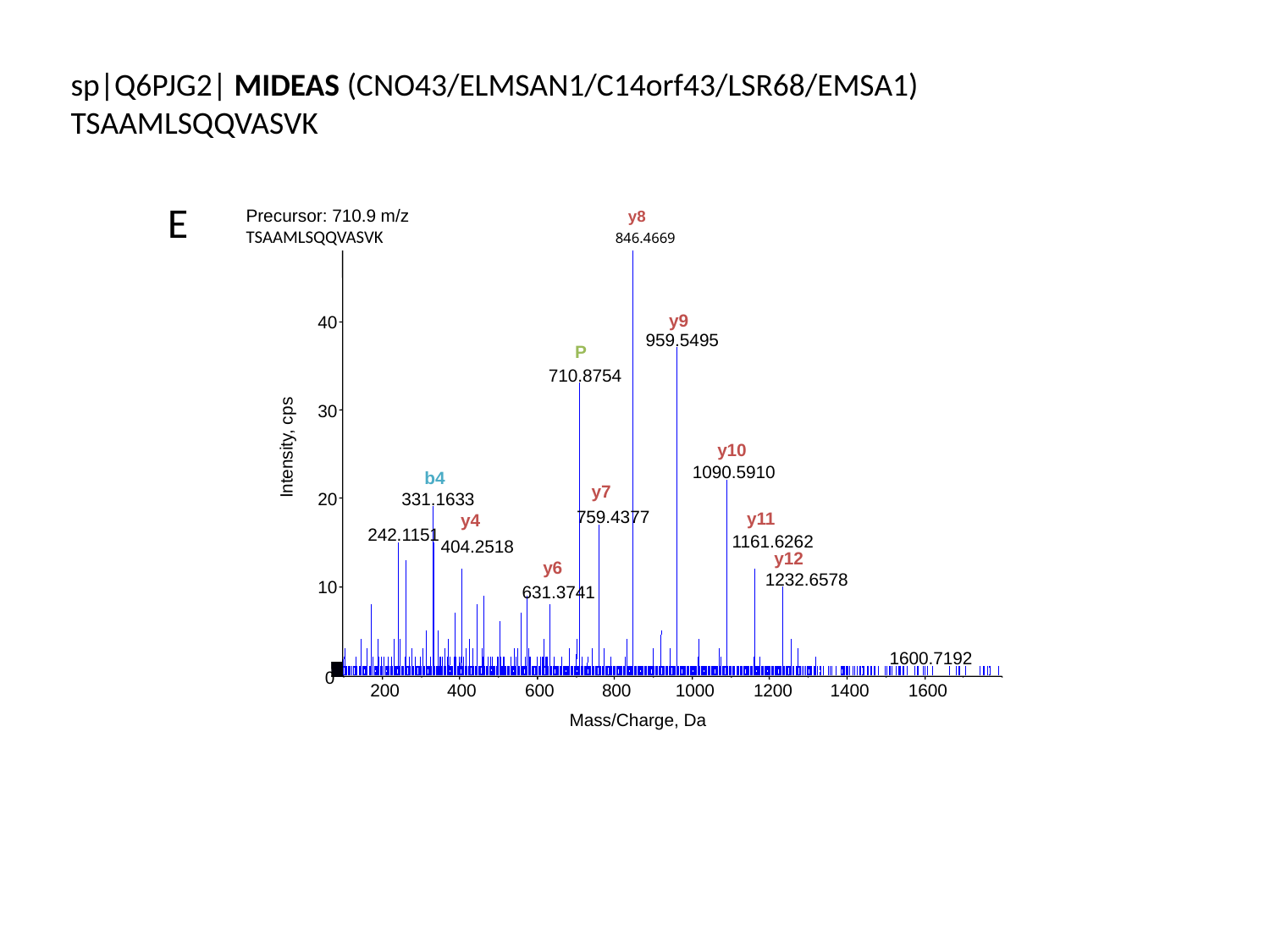

sp|Q6PJG2| MIDEAS (CNO43/ELMSAN1/C14orf43/LSR68/EMSA1)
TSAAMLSQQVASVK
E
y8
Precursor: 710.9 m/z
TSAAMLSQQVASVK
846.4669
y9
40
959.5495
P
710.8754
30
y10
Intensity, cps
b4
1090.5910
y7
20
331.1633
y11
y4
759.4377
242.1151
1161.6262
404.2518
y12
y6
1232.6578
10
631.3741
1600.7192
0
200
400
600
800
1000
1200
1400
1600
Mass/Charge, Da

## Slide 6
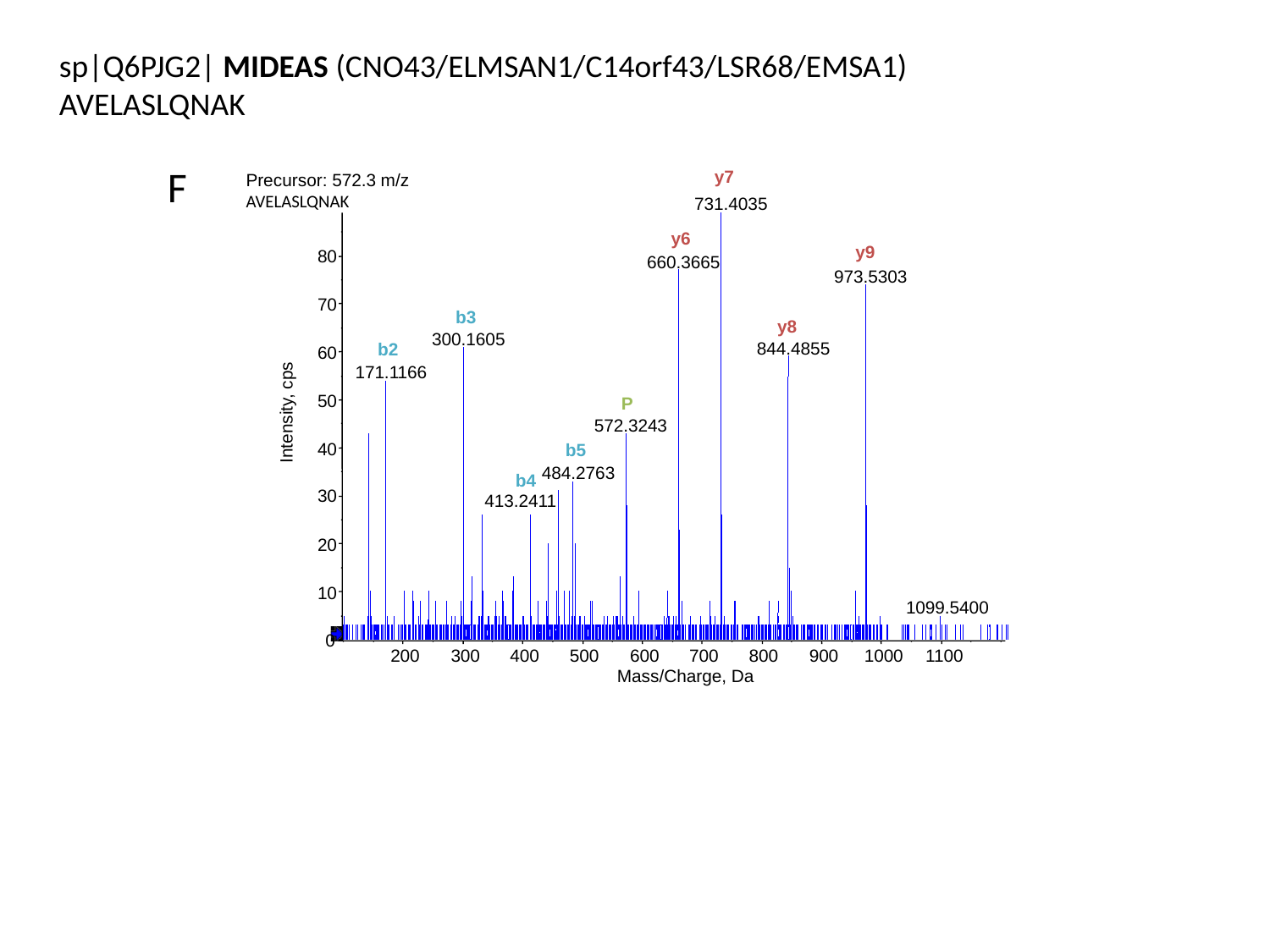

sp|Q6PJG2| MIDEAS (CNO43/ELMSAN1/C14orf43/LSR68/EMSA1)
AVELASLQNAK
F
y7
Precursor: 572.3 m/z
AVELASLQNAK
731.4035
y6
y9
80
660.3665
973.5303
70
b3
y8
300.1605
b2
844.4855
60
171.1166
P
50
Intensity, cps
572.3243
b5
40
484.2763
b4
30
413.2411
20
10
1099.5400
0
200
300
400
500
600
700
800
900
1000
1100
Mass/Charge, Da

## Slide 7
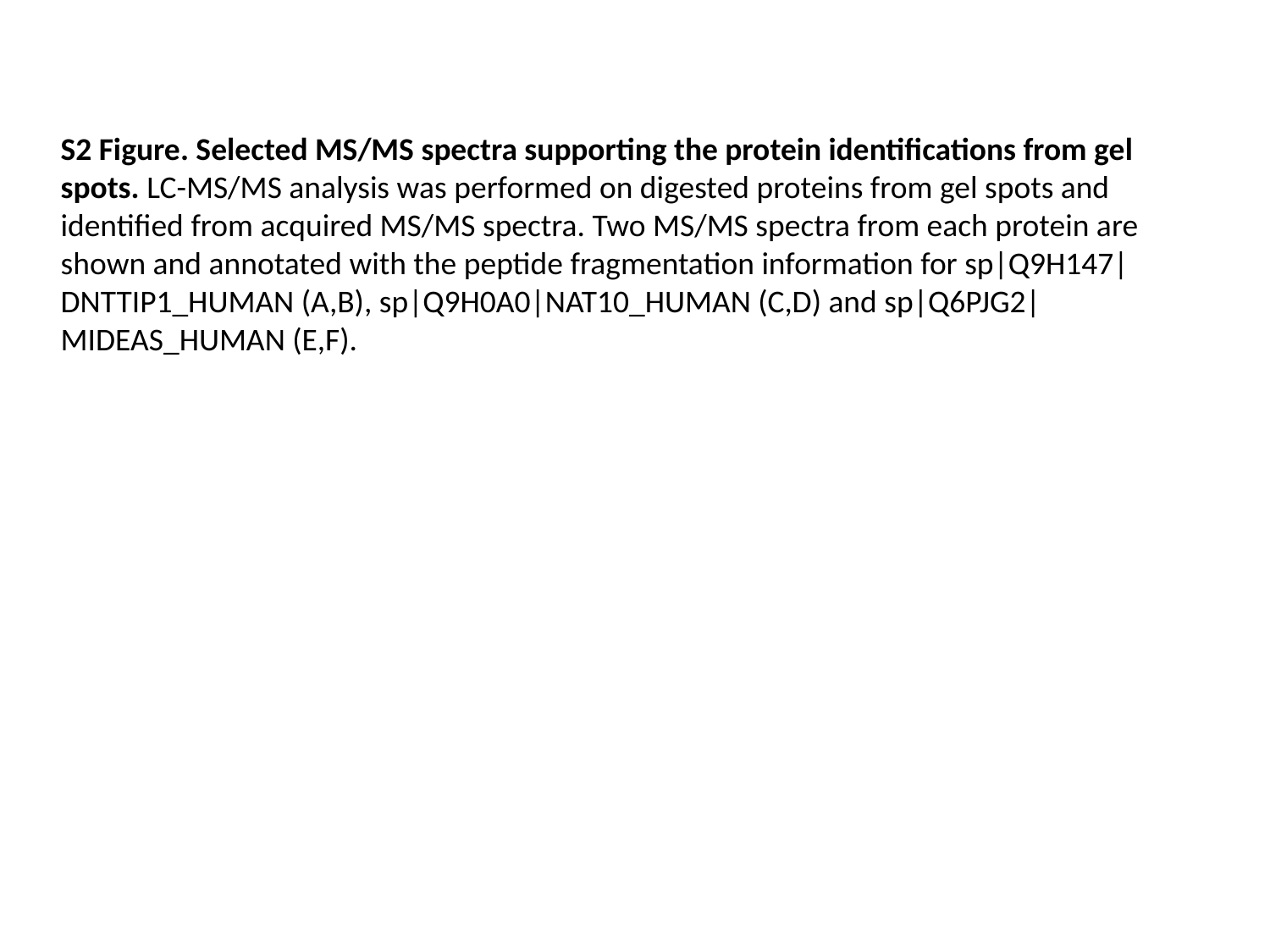

S2 Figure. Selected MS/MS spectra supporting the protein identifications from gel spots. LC-MS/MS analysis was performed on digested proteins from gel spots and identified from acquired MS/MS spectra. Two MS/MS spectra from each protein are shown and annotated with the peptide fragmentation information for sp|Q9H147|DNTTIP1_HUMAN (A,B), sp|Q9H0A0|NAT10_HUMAN (C,D) and sp|Q6PJG2|MIDEAS_HUMAN (E,F).
