# Supplementary material for: Mitotic deacetylase complex (MiDAC) recognizes the HIV-1 core promoter to control activated viral gene expression
Source: PLoS Pathog. 2024 May 23;20(5):e1011821. doi: 10.1371/journal.ppat.1011821 (PMC11115230; doi:10.1371/journal.ppat.1011821)
Supplement: S2 Table — (DOCX) [file ppat.1011821.s005.docx]

**S2 Table. Modulators of HIV transcription that act in part via the TATA-box region**

| **Modulator** * | **Impact on HIV transcription** | **Reference(s)** |
| --- | --- | --- |
| Sodium butyrate | Activation | [1] |
| TCERG1 (CA150) transcription elongation regulator 1 | Repression | [2] |
| Human herpesvirus 6 (HHV6) ORF-1 | Activation | [3] |
| Phorbol 12-myristate 13-acetate (PMA) or phytohemagglutinin A (PHA) † | Activation | [4] |
| PMA † | Activation | [5] |
| Pseudorabies virus immediate early gene (PIE) | Activation | [6] |
| Parvovirus B19 N  S1 protein | Activation | [7] |
| UV light †† | Activation (of stably integrated HIV reporter) | [8] |
| CD8 antiviral factor (CAF) | Repression | [9, 10] |
| Vpr HIV auxiliary protein | Activation | [11, 12] |

* Modulators that have been show to act via the broad mechanism of interaction with the general transcription factor TBP, for example adenovirus EIA [13] or CMV IE2 [14], have not been included in the table.

† The TATA box region can contribute to the PMA response in some reports; PMA is well known to act primarily via the NF-κB sites of the HIV LTR [15].

†† The NF-κB sites of the HIV LTR play a role in the UV response in some situations [16].

**References**

1. Golub EI, Li GR, Volsky DJ. Induction of dormant HIV-1 by sodium butyrate: involvement of the TATA box in the activation of the HIV-1 promoter. AIDS. 1991;5(6):663-8. PubMed PMID: 1883541.

2. Sune C, Garcia-Blanco MA. Transcriptional cofactor CA150 regulates RNA polymerase II elongation in a TATA-box-dependent manner. Mol Cell Biol. 1999;19(7):4719-28. Epub 1999/06/22. PubMed PMID: 10373521; PubMed Central PMCID: PMCPMC84270.

3. Kashanchi F, Thompson J, Sadaie MR, Doniger J, Duvall J, Brady JN, et al. Transcriptional activation of minimal HIV-1 promoter by ORF-1 protein expressed from the SalI-L fragment of human herpesvirus 6. Virology. 1994;201(1):95-106. Epub 1994/05/15. doi: 10.1006/viro.1994.1269. PubMed PMID: 8178493.

4. Li YC, Ross J, Scheppler JA, Franza BR, Jr. An in vitro transcription analysis of early responses of the human immunodeficiency virus type 1 long terminal repeat to different transcriptional activators. Mol Cell Biol. 1991;11(4):1883-93. PubMed PMID: 2005886.

5. Sakaguchi M, Zenzie-Gregory B, Groopman JE, Smale ST, Kim SY. Alternative pathway for induction of human immunodeficiency virus gene expression: involvement of the general transcription machinery. Journal of virology. 1991;65(10):5448-56. Epub 1991/10/01. PubMed PMID: 1895393; PubMed Central PMCID: PMC249034.

6. Yuan R, Bohan C, Shiao FC, Robinson R, Kaplan HJ, Srinivasan A. Activation of HIV LTR-directed expression: analysis with pseudorabies virus immediate early gene. Virology. 1989;172(1):92-9. Epub 1989/09/01. PubMed PMID: 2549725.

7. Sol N, Morinet F, Alizon M, Hazan U. Trans-activation of the long terminal repeat of human immunodeficiency virus type 1 by the parvovirus B19 NS1 gene product. J Gen Virol. 1993;74 ( Pt 9):2011-4. Epub 1993/09/01. doi: 10.1099/0022-1317-74-9-2011. PubMed PMID: 8376975.

8. Valerie K, Singhal A, Kirkham JC, Laster WS, Rosenberg M. Activation of human immunodeficiency virus gene expression by ultraviolet light in stably transfected human cells does not require the enhancer element. Biochemistry. 1995;34(48):15760-7. Epub 1995/12/05. PubMed PMID: 7495807.

9. Blazek D, Teque F, Mackewicz C, Peterlin M, Levy JA. The CD8+ cell non-cytotoxic antiviral response affects RNA polymerase II-mediated human immunodeficiency virus transcription in infected CD4+ cells. J Gen Virol. 2016;97(1):220-4. Epub 2015/10/27. doi: 10.1099/jgv.0.000326. PubMed PMID: 26499373; PubMed Central PMCID: PMCPMC4772706.

10. Shridhar V, Chen Y, Gupta P. The CD8 antiviral factor (CAF) can suppress HIV-1 transcription from the long terminal repeat (LTR) promoter in the absence of elements upstream of the CATATAA box. Virol J. 2014;11:130. Epub 2014/07/23. doi: 10.1186/1743-422X-11-130. PubMed PMID: 25048949; PubMed Central PMCID: PMCPMC4223764.

11. Felzien LK, Woffendin C, Hottiger MO, Subbramanian RA, Cohen EA, Nabel GJ. HIV transcriptional activation by the accessory protein, VPR, is mediated by the p300 co-activator. Proc Natl Acad Sci U S A. 1998;95(9):5281-6. Epub 1998/06/06. PubMed PMID: 9560267; PubMed Central PMCID: PMCPMC20252.

12. Gummuluru S, Emerman M. Cell cycle- and Vpr-mediated regulation of human immunodeficiency virus type 1 expression in primary and transformed T-cell lines. J Virol. 1999;73(7):5422-30. Epub 1999/06/11. PubMed PMID: 10364289; PubMed Central PMCID: PMCPMC112598.

13. Song CZ, Loewenstein PM, Toth K, Green M. Transcription factor TFIID is a direct functional target of the adenovirus E1A transcription-repression domain. Proc Natl Acad Sci U S A. 1995;92(22):10330-3. PubMed PMID: 7479778.

14. Hagemeier C, Walker S, Caswell R, Kouzarides T, Sinclair J. The human cytomegalovirus 80-kilodalton but not the 72-kilodalton immediate-early protein transactivates heterologous promoters in a TATA box-dependent mechanism and interacts directly with TFIID. J Virol. 1992;66(7):4452-6. Epub 1992/07/01. PubMed PMID: 1318414; PubMed Central PMCID: PMCPMC241253.

15. Nabel G, Baltimore D. An inducible transcription factor activates expression of human immunodeficiency virus in T cells. Nature. 1987;326(6114):711-3. PubMed PMID: 3031512.

16. Stein B, Rahmsdorf HJ, Steffen A, Litfin M, Herrlich P. UV-induced DNA damage is an intermediate step in UV-induced expression of human immunodeficiency virus type 1, collagenase, c-fos, and metallothionein. Mol Cell Biol. 1989;9(11):5169-81. Epub 1989/11/01. PubMed PMID: 2557547; PubMed Central PMCID: PMCPMC363669.
