# Supplementary material for: Mitotic deacetylase complex (MiDAC) recognizes the HIV-1 core promoter to control activated viral gene expression
Source: PLoS Pathog. 2024 May 23;20(5):e1011821. doi: 10.1371/journal.ppat.1011821 (PMC11115230; doi:10.1371/journal.ppat.1011821)
Supplement: S3 Table — (DOCX) [file ppat.1011821.s006.docx]

**S3 Table. Potential links of DNTTIP1, MIDEAS & NAT10 to HIV biology**

| **Protein** | **Proteomic, genomic, or clinical data** | **Reference(s)** |
| --- | --- | --- |
| **DNTTIP1** | mRNA upregulated in CD4+ cells infected by Env/Nef deficient HIV-1 viruses versus those infected with HIV-1 viruses lacking Env, Nef and Vpr | [1] |
|  | mRNA upregulated 12 hours after HIV infection of SUP-T1 T cells | [2] |
|  | mRNA under-represented in the CD4+ cells of HIV resistant cohort of sex workers versus HIV negative control subjects | [3] |
| **MIDEAS** | mRNA overrepresented in the CD3+ cells of long term non-progressors (LTNP) with respect to progressors | [4] |
|  | mRNA overrepresented in the CD8+ cells of non-progressors vs early and chronic HIV infections | [5] |
|  | mRNA induced in dendritic cells treated with Pr55gag Virus-Like Particles (HIV-VLPs) | [6] |
| **NAT10** | Interaction with HIV Rev protein | [7] |
|  | Interaction with HIV Gag protein | [8-10] |
|  | Altered nuclear distribution during HIV infection | [11] |
|  | Interaction with HIV Tat *trans*-activating protein | [12, 13] |

**References**

1. Dabrowska A, Kim N, Aldovini A. Tat-induced FOXO3a is a key mediator of apoptosis in HIV-1-infected human CD4+ T lymphocytes. J Immunol. 2008;181(12):8460-77. Epub 2008/12/04. PubMed PMID: 19050264; PubMed Central PMCID: PMCPMC2665797.

2. Chang S, Sova P, Aicher L, Katze MG. Database: GEO [Internet]. Available from: <https://www.ncbi.nlm.nih.gov/geo/query/acc.cgi?acc=GSE56484>. 2014.

3. McLaren PJ, Ball TB, Wachihi C, Jaoko W, Kelvin DJ, Danesh A, et al. HIV-exposed seronegative commercial sex workers show a quiescent phenotype in the CD4+ T cell compartment and reduced expression of HIV-dependent host factors. J Infect Dis. 2010;202 Suppl 3:S339-44. Epub 2010/10/05. doi: 10.1086/655968. PubMed PMID: 20887221.

4. Salgado M, Lopez-Romero P, Callejas S, Lopez M, Labarga P, Dopazo A, et al. Characterization of host genetic expression patterns in HIV-infected individuals with divergent disease progression. Virology. 2011;411(1):103-12. Epub 2011/01/18. doi: 10.1016/j.virol.2010.12.037. PubMed PMID: 21239032.

5. Hyrcza MD, Kovacs C, Loutfy M, Halpenny R, Heisler L, Yang S, et al. Distinct transcriptional profiles in ex vivo CD4+ and CD8+ T cells are established early in human immunodeficiency virus type 1 infection and are characterized by a chronic interferon response as well as extensive transcriptional changes in CD8+ T cells. J Virol. 2007;81(7):3477-86. Epub 2007/01/26. doi: 10.1128/JVI.01552-06. PubMed PMID: 17251300; PubMed Central PMCID: PMCPMC1866039.

6. Arico E, Wang E, Tornesello ML, Tagliamonte M, Lewis GK, Marincola FM, et al. Immature monocyte derived dendritic cells gene expression profile in response to Virus-Like Particles stimulation. Journal of translational medicine. 2005;3:45. Epub 2005/12/31. doi: 10.1186/1479-5876-3-45. PubMed PMID: 16384534; PubMed Central PMCID: PMC1360684.

7. Naji S, Ambrus G, Cimermancic P, Reyes JR, Johnson JR, Filbrandt R, et al. Host cell interactome of HIV-1 Rev includes RNA helicases involved in multiple facets of virus production. Mol Cell Proteomics. 2012;11(4):M111 015313. Epub 2011/12/17. doi: 10.1074/mcp.M111.015313. PubMed PMID: 22174317; PubMed Central PMCID: PMCPMC3322577.

8. Engeland CE, Brown NP, Borner K, Schumann M, Krause E, Kaderali L, et al. Proteome analysis of the HIV-1 Gag interactome. Virology. 2014;460-461:194-206. Epub 2014/07/11. doi: 10.1016/j.virol.2014.04.038. PubMed PMID: 25010285.

9. Engeland CE, Oberwinkler H, Schumann M, Krause E, Muller GA, Krausslich HG. The cellular protein lyric interacts with HIV-1 Gag. J Virol. 2011;85(24):13322-32. Epub 2011/10/01. doi: 10.1128/JVI.00174-11. PubMed PMID: 21957284; PubMed Central PMCID: PMCPMC3233182.

10. Le Sage V, Cinti A, Valiente-Echeverria F, Mouland AJ. Proteomic analysis of HIV-1 Gag interacting partners using proximity-dependent biotinylation. Virol J. 2015;12:138. Epub 2015/09/13. doi: 10.1186/s12985-015-0365-6. PubMed PMID: 26362536; PubMed Central PMCID: PMCPMC4566291.

11. DeBoer J, Jagadish T, Haverland NA, Madson CJ, Ciborowski P, Belshan M. Alterations in the nuclear proteome of HIV-1 infected T-cells. Virology. 2014;468-470:409-20. Epub 2014/09/23. doi: 10.1016/j.virol.2014.08.029. PubMed PMID: 25240327; PubMed Central PMCID: PMCPMC4253593.

12. Gautier VW, Gu L, O'Donoghue N, Pennington S, Sheehy N, Hall WW. In vitro nuclear interactome of the HIV-1 Tat protein. Retrovirology. 2009;6:47. Epub 2009/05/21. doi: 10.1186/1742-4690-6-47. PubMed PMID: 19454010; PubMed Central PMCID: PMC2702331.

13. Jean MJ, Power D, Kong W, Huang H, Santoso N, Zhu J. Identification of HIV-1 Tat-Associated Proteins Contributing to HIV-1 Transcription and Latency. Viruses. 2017;9(4). doi: 10.3390/v9040067. PubMed PMID: 28368303; PubMed Central PMCID: PMCPMC5408673.
