# Supplementary material for: Mitotic deacetylase complex (MiDAC) recognizes the HIV-1 core promoter to control activated viral gene expression
Source: PLoS Pathog. 2024 May 23;20(5):e1011821. doi: 10.1371/journal.ppat.1011821 (PMC11115230; doi:10.1371/journal.ppat.1011821)
Supplement: S1 File — The file contains: 1) Oligonucleotides and target sequences; 2) DNTTIP1 purification dialysis buffers and protocol; 3) Latency Reactivating Agents used in the study. (DOCX) [file ppat.1011821.s007.docx]

**S1 File. Additional material and methods**

**1) Oligonucleotides and target sequences.**

| **Name** | **Sequence 5'=> 3'** | **Application** |
| --- | --- | --- |
| **TDIF1-BamL** | GATCCATGGGAGCCACTGGCGA | pcDNA3.1 subcloning |
| **TDIF1-BamC** | CATGGGAGCCACTGGCGACG |  |
| **TDIF1-XhoL** | TCGAGTCAGGTCTGTGGAGGTGCTTC | pcDNA3.1 and plenti 6V5 subcloning |
| **TDIF1-XhoC** | GTCAGGTCTGTGGAGGTGCTTC |  |
| **TDIF1-Flag-EcoRI_Lg** | AATTCTTACTTATCGTCGTCATCCTTGTAATCGGTCTGTGGAGGTGCTTCAAC | plenti 6V5 subcloning with a C-terminal Flag tag |
| **TDIF1-Flag-EcoRI_Ct** | CTTACTTATCGTCGTCATCCTTGTAATCGGTCTGTGGAGGTGCTTCAAC |  |
| **pNL ATACq+** | ATACATATAAGTATATACTTTTTGCCTGTACTGGGTCTCTCTGG | CTGC to ATAC directed mutagenesis of all three motifs in HIV 5'LTR core promoter |
| **pNL ATACq-** | TATATACTTATATGTATCATCTGAGGGCTCGCCACTCC |  |
| **pNL mkB+** | GCTACAACTCACTTTCCGCTGCTCACTTTCCAGGGAGGC | directed mutagenesis of NF-κB sites in HIV 5'LTR |
| **pNL mkB-** | GAGCAGCGGAAAGTGAGTTGTAGCAAGCTCGATGTCAGCAGTTCTTGA |  |

**Table A. Sub-cloning primers**

| **Name** | **Sequence 5'=> 3'** | **Target** |
| --- | --- | --- |
| **ChIP_HIV-455_f** | GGAAGGGCTAATTCACTCCCAA | HIV LTR : -455 upstream of TSS |
| **ChIP_HIVprom-455_r** | CCTGGTGTGTAGTTCTGCCAA | HIV LTR : -356 upstream of TSS |
| **ChIP_HIVprom-90_f** | GACTTTCCAGGGAGGCGTG | HIV LTR : -90 upstream of TSS |
| **HIVprom-** | AGGCTCAGATCTGGTCTAACCAGAGA | HIV LTR: +29 downstream of TSS |
| **ChIP_DDIT3prom_f** | CGTGCCACTTTCTGATTGGT | ddit3 gene promoter |
| **ChIP_DDIT3prom_r** | GGTTCCAGCTCTGATTTTGG |  |
| **ChIP_bGlobprom_f** | AGGGCTGAGGGTTTGAAGTCCAACTC | β-Globine gene promoter |
| **ChIP_bGlobprom_r** | TGTAAGCAATAGATGGCTCTGCCCTGAC |  |
| **ChIP_GDM_f** | AAACAAGCATCAGGGTGGAC | intergenic desert in chromosom 16 |
| **ChIP_GDM_r** | GATCCCACAAAGGAAAGGAAC |  |

**Table B. Primers used for ChIP qPCR**

| **Name** | **Sequence 5'=> 3'** |
| --- | --- |
| **HIV-wt+** | CTAGATGCTGCATATAAGCAGCTGCTTTTTGCATG |
| **BIOTeg-HIVwt-** | Biotin-Teg-CATGCAAAAAGCAGCTGCTTATATGCAGCAT |
| **HIV-ATCC+** | CTAGATG**A**T**C**CATATAAG**G**A**TA**T**C**CTTTTTGCATG |
| **BIOTeg-HIV-ATCC-** | Biotin-Teg-CATGCAAAAAG**G**A**TA**T**C**CTTATATG**G**A**T**CAT |
| **HIV-ATAC+** | CTAGATG**A**T**A**CATATAAG**T**A**TA**T**A**CTTTTTGCATG |
| **BIOTeg-HIV-ATAC-** | Biotin-Teg-CATGCAAAAAG**T**A**TA**T**A**CTTATATG**T**A**T**CAT |
| **HIV-CATAKO+** | CTAGATGCTG**AGAGCTC**GCAGCTGCTTTTTGCATG |
| **BIOTeg-HIV-CATAKO-** | Biotin-Teg-CATGCAAAAAGCAGCTGC **GAGCTCT**CAGCAT |
| **HIV-USF1KO+** | CTAGATGCTGCATATAAGCA**CG**TGCTTTTTGCATG |
| **BIOTeg- HIV-USF1KO-** | Biotin-Teg-CATGCAAAAAGCA**CG**TGC TTATATGCAGCAT |
| **HIV-USF1+ +** | CTAGATGCTGCATATAAGC**G**GC**C**GCTTTTTGCATG |
| **BIOTeg- HIV-USF1+ -** | Biotin-Teg-CATGCAAAAAGC**G**GC**C**GC TTATATGCAGCAT |
| **Scr+** | CTAGA**GTAGCAGAGCTCTGGTAGCTAGAAA**GCATG |
| **BIOTeg- Scr-** | Biotin-Teg-CATGC**TTTCTAGCTACCAGAGCTCTGCTAC**T |
| **Oligo B+** | GATCCGGAGTACTTCAAGAACG |
| **Oligo B-** | GATCCGTTCTTGAAGTACTCCG |

**Table C. Oligonucleotides used for affinity chromatography.**

« Teg " stands for a triethyleneglycol spacer inserting 15 atomes between the biotine molecule and the nucleotide sequence. Sense (+) and reverse (-) oligonucleotides were annealed before use.

| **Name** | **Sequence 5'=> 3'** | **Application** |
| --- | --- | --- |
| **TASHET_25+** | AGA**T**GCTGCATATAAGCAGCTGCTT | CD / Fluo aniso |
| **TASHET_25-** | AAGCAGCTGCTTATATGCAGCATCT | CD / Fluo aniso |
| **ATCC_25+** | AGA**T**GATCCATATAAGGATATCCTT | CD / Fluo aniso |
| **ATCC_25-** | AAGGATATCCTTATATGGATCATCT | CD / Fluo aniso |
| **DNTTIP_cons+** | CGCCAAATAAAAGTTGCATGGTCG | CD |
| **DNTTIP_cons-** | CGACCATGCAACTTTTATTTGGCG | CD |
| **CATAKO_25+** | AGA**T**GCTGAGAGCTCGCAGCTGCTT | Fluo aniso |
| **CATAKO_25-** | AAGCAGCTGCGAGCTCTCAGCATCT | Fluo aniso |
| **Scr_25+** | AGA**T**CAGCAGAGCTCTGGTAGCTAT | Fluo aniso |
| **Scr_25-** | ATAGCTACCAGAGCTCTGCTGATCT | Fluo aniso |

**Table D. Oligonucleotides used in ciruclar dichroïsm (CD) and fluorescence anisotropy (Fluo aniso).**

Sequences are identical for both application with the coupling of a fluorescein molecule to the first thymine (in green) on the sens oligo for Fluorescence anisotropy. Sense (+) and reverse (-) oligonucleotides were annealed before use.

| **Name** | **Target sequence** | **Target** |
| --- | --- | --- |
| **D1** | 5'-CAGACTAGCATGTGGTTCTATATTT-3' | NM_052951.2 ; 3'UTR of dnttip1 gene |
| **D2** | 5'-CGCCGCTCACAGATGACAACAAGT T-3' | NM_052951.2 ; exon 2-3 of dnttip1 gene |
| **M1** | 5'-CCGAGAGGAAGCTGTTCAACAAAGG-3' | NM_194278 ; exon 8 of c14orf43 gene |
| **M2** | 5'-GCCGCAGTGCGCATATGAAGAGCCA-3' | NM_194278 ; 3'UTR of c14orf43 gene |
| **M3** | 5'-GGCGACTGTTGAAGGGGATCTCACC-3' | NM_194278 ; 5'UTR of c14orf43 gene |
| **N1** | 5'-CAACATCACTCGGATAGTCTCAGGC-3' | NM_024662 |
| **N2** | 5'-CCTGTAGTTTATGTAGAATGCCACA-3' | NM_024662 ; 3'UTR of nat10 gene |
| **Ctrl-** | 5'-CTTCCTCTCTTTCTCTCCCTTGTGA-3' | non target ctrl : ref#51-01-14-03 IDT-DNA |

**Table E. dsiRNA targeting sequences.**

The sense sequence targeted by the dsiRNA are displayed.

| **Name** | **Ref** | **Sequence 5'=> 3'** | **Target** |
| --- | --- | --- | --- |
| **Ctrl** | SHC202 | CCGG**CAACAAGATGAAGAGCACCAA**CTCGAG**TTGGTGCTCTTCATCTTGTTG**TTTTT | non mammalian |
| **#1** | TRCN0000365477 | CCGG**TTACGAACCCTTGGAACATAA**CTCGAG**TTATGTTCCAAGGGTTCGTAA**TTTTTG | human DNTTIP1 |
| **#2** | TRCN0000365476 | CCGG**TCAAGCACCCACACCTCTTTA**CTCGAG**TAAAGAGGTGTGGGTGCTTGA**TTTTTG | human DNTTIP1 |
| **#3** | TRCN0000370729 | CCGG**CACTGAACGTGCGAGACAATG**CTCGAG**CATTGTCTCGCACGTTCAGTG**TTTTTG | human DNTTIP1 |

**Table F. TRC2 sh RNA lentiviral constructs used in the study.**

shRNA constructs targeting DNTTIP1 are Sigma-Aldrich product type : SHCLNG-NM_052951. The specific nucleotides composing the hairpin are in bold characters.

**2) DNTTIP1 purification dialysis buffers.**

The purified His-tagged DNTTIP1 protein was dialysed in suitable buffers for each of the following experiments:

1) CD spectrum : O/N dialysis against 200 volumes of Dial buffer I (25mM Na.PO_4_ pH6.5, 150mM KCl, 50mM L-Arg, 50mM L-Glu, 1mM DTT)

2) CD denaturation experiments : O/N dialysis against 200 volumes of Dial buffer CD (10mM Na.PO_4_ pH6.5, 1mM TCEP).

3) Fluorescence anisotropy : the purified protein was dialysed stepwise with decreasing concentration of KCl and Imidazole in Dial buffer II (10mM Na.PO_4_, 500mM to 150mM KCl, 5% glycerol, 150mM to no Imidazole, 1mM DTT) for 1h to O/N as detailed in the following table.

| **Buffer #** | **time** | **KCl** | **Imidazole pH8** | **DTT/TCEP** |
| --- | --- | --- | --- | --- |
| 1 | 1h | 500 mM | 150 mM | 1mM DTT |
| 2 | 1h | 250 mM | 75 mM | - |
| 3 | O/N | 150 mM | 25 mM | - |
| 4 | 1h | 150 mM | - | - |
| 5 | 1h | 150 mM | - | 250µM TCEP |

**Table G. DNTTIP1 dialysis timeline and buffer composition for fluorescence anisotropy**

All buffers contained 10mM Na.PO_4_ and 5% glycerol. Dialyses were performed at 4°C against a minimum of 100 volumes of buffer.

**3) Latency Reactivating Agents (LRA) used in the study.**

| **Product name** | **Supplier** | **Reference** | **Concentration** |
| --- | --- | --- | --- |
| **HMBA** | Sigma | 224235-10G | 3mM |
| **(+)-JQ1** | Cayman Chemical* | 11187 | 1µM [[1](#_ENREF_1)] |
| **MMQO[**[**2**](#_ENREF_2)**]** | Pr Albert Jordan** | [[2](#_ENREF_2)] | 100µM |
| **Prostratin** | Cayman Chemical | 10272 | 1.25µM [[3](#_ENREF_3)] |
| **Romidepsin** | Cayman Chemical | 17130 | 40nM [[4](#_ENREF_4), [5](#_ENREF_5)] |
| **SAHA** | Sigma | SML0061 | 1µM |
| **Recombinant human TNF-α** | Novus Biologicals*** | NBP2-35076-10ug | 10ng/ml |

**Table H. Latency Reversing Agents (LRA) used in the study.**

* Cayman Chemical (Ann Harbor, MI, USA) ; ** MMQO (8-methoxy-6-

methylquinolin-4-ol) was kindly provided by Pr. Albert Jordan (Institut de Biologia Molecular de Barcelona (IBMB-CSIC), Barcelona, Espagne) ; *** Novus Biologicals (Littleton, CO, USA). The concentration choice was based on indicated litterature and on several comparative reactivation studies [[6-8](#_ENREF_6)].

**References**

1. Li Z, Guo J, Wu Y, Zhou Q. The BET bromodomain inhibitor JQ1 activates HIV latency through antagonizing Brd4 inhibition of Tat-transactivation. Nucleic Acids Res. 2013;41(1):277-87. Epub 2012/10/23. doi: 10.1093/nar/gks976. PubMed PMID: 23087374; PubMed Central PMCID: PMC3592394.

2. Gallastegui E, Marshall B, Vidal D, Sanchez-Duffhues G, Collado JA, Alvarez-Fernandez C, et al. Combination of biological screening in a cellular model of viral latency and virtual screening identifies novel compounds that reactivate HIV-1. J Virol. 2012;86(7):3795-808. Epub 2012/01/20. doi: 10.1128/JVI.05972-11. PubMed PMID: 22258251; PubMed Central PMCID: PMC3302487.

3. Bedoya LM, Marquez N, Martinez N, Gutierrez-Eisman S, Alvarez A, Calzado MA, et al. SJ23B, a jatrophane diterpene activates classical PKCs and displays strong activity against HIV in vitro. Biochem Pharmacol. 2009;77(6):965-78. Epub 2008/12/23. doi: 10.1016/j.bcp.2008.11.025. PubMed PMID: 19100719.

4. Bantscheff M, Hopf C, Savitski MM, Dittmann A, Grandi P, Michon AM, et al. Chemoproteomics profiling of HDAC inhibitors reveals selective targeting of HDAC complexes. Nat Biotechnol. 2011;29(3):255-65. PubMed PMID: 21258344.

5. Wei DG, Chiang V, Fyne E, Balakrishnan M, Barnes T, Graupe M, et al. Histone deacetylase inhibitor romidepsin induces HIV expression in CD4 T cells from patients on suppressive antiretroviral therapy at concentrations achieved by clinical dosing. PLoS Path. 2014;10(4):e1004071. Epub 2014/04/12. doi: 10.1371/journal.ppat.1004071. PubMed PMID: 24722454; PubMed Central PMCID: PMC3983056.

6. Spina CA, Anderson J, Archin NM, Bosque A, Chan J, Famiglietti M, et al. An in-depth comparison of latent HIV-1 reactivation in multiple cell model systems and resting CD4+ T cells from aviremic patients. PLoS Path. 2013;9(12):e1003834. Epub 2014/01/05. doi: 10.1371/journal.ppat.1003834. PubMed PMID: 24385908; PubMed Central PMCID: PMC3873446.

7. Darcis G, Kula A, Bouchat S, Fujinaga K, Corazza F, Ait-Ammar A, et al. An In-Depth Comparison of Latency-Reversing Agent Combinations in Various In Vitro and Ex Vivo HIV-1 Latency Models Identified Bryostatin-1+JQ1 and Ingenol-B+JQ1 to Potently Reactivate Viral Gene Expression. PLoS Path. 2015;11(7):e1005063. Epub 2015/08/01. doi: 10.1371/journal.ppat.1005063. PubMed PMID: 26225566; PubMed Central PMCID: PMC4520688.

8. Laird GM, Bullen CK, Rosenbloom DI, Martin AR, Hill AL, Durand CM, et al. Ex vivo analysis identifies effective HIV-1 latency-reversing drug combinations. The Journal of clinical investigation. 2015;125(5):1901-12. Epub 2015/03/31. doi: 10.1172/JCI80142. PubMed PMID: 25822022; PubMed Central PMCID: PMC4463209.
