# Supplementary material for: Mitotic deacetylase complex (MiDAC) recognizes the HIV-1 core promoter to control activated viral gene expression
Source: PLoS Pathog. 2024 May 23;20(5):e1011821. doi: 10.1371/journal.ppat.1011821 (PMC11115230; doi:10.1371/journal.ppat.1011821)
Supplement: Attachment — Submitted filename: Response to Reviewers PPAT-D-23_01994.docx [file ppat.1011821.s008.docx]

| Faculté de médecine et des sciences  de la santé (FMSS)  Pavillon de Recherche Appliquée sur le Cancer (PRAC)  3201, Rue Jean-Migneault  Sherbrooke (Québec)  CANADA J1E 4K8 | Brendan Bell, Ph.D.  Département de Microbiologie et infectiologie  Téléphone : (819) 821-8000 Poste 75327  Fax : (819) 820-6831  e-mail : [Brendan.Bell@USherbrooke.ca](mailto:Brendan.Bell@USherbrooke.ca) |
| --- | --- |

March 31, 2024

#### Dear Dr. Emerman,

Thank you for your time and expertise as a guest editor in the evaluation of our manuscript (PPATHOGENS-D-23-01994) now entitled “Mitotic deacetylase complex (MiDAC) recognizes the HIV-1 core promoter to control activated viral gene expression”. We also thank the three reviewers for their comments and questions. We were encouraged to read that globally there is a consensus that the findings are of potentially high significance and quality. We have uploaded a revised version after careful consideration of the evaluations and after having performed experiments suggested by the reviewers. As I think everyone can appreciate, not all the experimental suggestions can be fulfilled within 60 days, although there are many excellent ideas to be explored. Our conviction is that with the improvements and clarifications, the work is now ready to be published in a highly respected and highly visible platform, *PLOS Pathogens*, allowing the broader research community to build upon it collectively.

As suggested, we have removed any claims about HIV latency and replaced them with activation of HIV gene expression. The term latency reversing agent (LRA) remains only because that is the most commonly and therefore recognizably used term in the literature. In the sections below, we address each of the reviewers’ comments specifically.

**Part I - Summary**
Please use this section to discuss strengths/weaknesses of study, novelty/significance, general execution and scholarship.

Reviewer #1: This well-written manuscript from Wilhelm and colleagues investigates cellular proteins that can bind to the HIV-1 promoter (termed TASHET by Authors) and function to contribute to RNAP II and HIV-1 Tat transcription of the integrated viral genome. The proteins DNTTIP1, MIDEAS, and NAT10 were identified by virtue of their specific binding to the viral promoter and not mutant viral promoters. DNTTPIP1 and MIDEAS are subunits of a HDAC complex termed MiDAC whose activity increases during mitosis. NAT10, an acetyltransferase, has previously been reported to bind to the Tat protein. SiRNA depletions indicate that depletion of the three proteins individually can reduce expression from the viral LTR.

The Authors present clear evidence that DNTTIP1, MIDEAS, and NAT10 associate specifically with the viral promoter in vitro, and that DNTTIP1 does so in cells. They also present convincing evidence from their siRNA depletions that these factors can function to enhance HIV-1 LTR expression. However, the effects of siRNA depletions on a reporter plasmid are rather modest – generally, only a few fold at best. As described below, the “latency” reactivation experiment presented in Figure 6 is not up to the standard of the field. Also as described below, the impact of the study is limited without additional experiments from primary CD4+ T cells and patient cells harboring latent HIV-1.

Reviewer #2: Wilhem and colleagues use biotinylated oligonucleotides containing the WT TASHET sequence of HIV as determined by an earlier paper of the Bell group as well as oligos containing mutations within the GTGC motifs of TASHET to enrich for binding proteins from HeLa, Jurkat, and total PBMC extracts. Mass spec to identify proteins was specifically performed on material enriched from HeLa extracts and selective band excision based on visually identifiable differences was used rather than complex mixture, in solution analysis which may have resulted a more unbiased approach. They identified MIDEAS and DNTTIP1, and to a weaker extent NAT10, as novel binders of the TASHET sequence, members of the mitotic deacetylase complex (MiDAC).
The follow-up experiments to confirm binding to the oligos in question are well done, however the overall levels of enrichment observed by western blot are a bit underwhelming but appear specific. A stronger, validated binder such as TBP would been good to see as a positive control in the oligo enrichment experiments. The ChIP is well done with appropriate controls. We have concerns about appropriate controls in the later experiments and are unclear as to the proposed mechanism and when MiDAC functions in the HIV lifecycle as most experiments are undertaken with single round infections after 48hrs. We would like to see the points below addressed.

With respect to TBP, it does not bind detectably under our conditions in the DNA-affinity chromatography. We optimized the conditions for these assays over a period of years and the most suitable positive control is AP4 whose is known to bind the LTR (PMID: 16540471; 22834489) and whose level of enrichment is seen in Figure 2A, lane 2.

Reviewer #3: In this manuscript Wilhelm et al., identify and characterize a novel complex which binds the TATA box and adjacent sequences of the HIV-1 LTR essential for Tat trans-activation (TASHET). For this, they used sequence-specific DNA affinity chromatography where they purified a complex containing the factors DNTTIP1, MIDEAS, and NAT10. MIDEAS and DNTTIP represent components of the MiDAC histone deacetylase complex which is primarily active during mitosis. NAT10 is an acetyltransferase which was previously shown to interact with the HIV-1 viral transactivator Tat. Binding of this complex with the HIV-1 core promoter was shown to be dependent upon nucleotides near the TATA element required for responsiveness to Tat. Using HeLa cells, they demonstrated stronger overall binding of this complex with TASHET in cells arrested in mitosis, compared to an unsynchronized cell population. Using co-immunoprecipitation, they demonstrate that these proteins are associated in the absence of DNA, and that NAT10, at least also interacts with Cyclin T1. They used several biophysical techniques to determine that recombinant DNTTIP1 was capable of specifically binding TASHET DNA oligos in vitro. Using chromatin immunoprecipitation, they showed that Flag-tagged DNTTIP1 also bound the HIV-1 LTR in HeLa cells. Knocking down expression of these proteins in HeLa cells with siRNA inhibited HIV-1 expression in HeLa cells, both in the presence and absence of Tat. The largest effect is produced by DNTTIP1 and NAT10 siRNAs. They also examined the effect of shRNAs against DNTTIP1 on HIV-1 expression in the MOLT4 T cell line, where they observe ~50-60% reduced HIV-1 expression with three different shRNAs. Mutations that prevent binding of DNTTIP1 to the LTR, and inhibit activation by TAT, were also shown to inhibit reactivation by BET inhibitors, which are thought to work by encouraging release of P-TEFb from 7SK.

This is a very interesting report which employs some elegant biochemistry to identify a novel complex that interacts with sequences at the core HIV-1 LTR promoter required for activation by viral Tat. Association of this complex with this region of the LTR raises several important questions. First, many other proteins and complexes are known to bind this same region, not the least of which are the GTFs, RNA PolII, mediator, TFIID etc, but also numerous others, including YY1, LSF, AP4, ZASC1, and USF1/2. What seems to be unique about this complex is that its affinity towards the TASHET sequence is apparently stronger in cells arrested in mitosis. As such this manuscript would be strengthened considerably by a more detailed analysis of interaction with the LTR in vivo during the cell cycle. I don't believe anyone has examined effects of cell cycle on regulation of HIV-1 transcription, or TAT transactivation, and such an analysis would make this a very exciting study. Relating to this, strangely, I note that the authors don't really discuss the potential role of cell cycle for HIV-1 transcriptional regulation in the manuscript. Secondly, this putative complex contains both an HDAC and an acetyltransferase; the authors mention in the Discussion that acetylation/ deacetylation dynamics on histones may contribute to control of transcriptional elongation. For this manuscript it would be important to demonstrate the extent that the HDAC and acetylase activities are required for their effect on HIV-1 expression, and their effect on modification of histones at the LTR.

We share with the reviewer a fascination for the relationship between the cell cycle and HIV transcription. Based on the comments, we initiated and optimized protocols to test the impact of mutations that block the binding of DNTTIP1 to the TATA-box region on cell cycle regulation of the LTR. After a significant amount of work, the data show that the induction of cell cycle arrest at G2/M with nocodazole increases HIV transcription, as has been previously reported, but that the CTGC motifs are dispensable for the induction. On the other hand, we have very preliminary data hinting that HIV Vpr stimulation of the LTR does require the CTGC motifs.

Until we have a clear idea of the mechanistic links between cell cycle, MiDAC and gene expression, claims about the relative importance of MiDAC would be premature. The first compelling report of HIV expression being controlled by the cell cycle was published 26 years ago in 1998 (PMID: 9427608). It will take years to fully unravel the underlying mechanisms, but the identification of MiDAC as controlling the HIV LTR will undoubtedly accelerate the process.

**Part II – Major Issues: Key Experiments Required for Acceptance**
Please use this section to detail the key new experiments or modifications of existing experiments that should be absolutely required to validate study conclusions.

Generally, there should be no more than 3 such required experiments or major modifications for a "Major Revision" recommendation. If more than 3 experiments are necessary to validate the study conclusions, then you are encouraged to recommend "Reject".

Reviewer #1: 1. As described in Methods, the “latency reversal” experiment presented in Figure 7 involved an infection of Jurkat cells for 36 hours, followed by addition of LRAs. It is not possible to distinguish effects of the LRAs on ongoing viral replication vs latency, and latency is likely to be limited at 36 hours. There are Jurkat lines available with latent virus and reporter proteins that available for this type of experiment with mutant LTRs.

There are many cellular latency models available, each with their own strengths and weaknesses, none being perfect. In our case, because we are studying novel DNA-binding proteins, we needed a system into which we could introduce mutations that are not available in any other laboratory. Furthermore, have removed discussions of latency and speak only of the activation of HIV gene expression in the new version of the manuscript.

2. The impact of the study would be increased if the levels of DNTTIP1, MIDEAS, and NAT10 were examined in resting and activated primary CD4+ T cells. This may demonstrate some interesting regulation of these proteins.

We share the reviewer’s interest in activated primary cells. We have tried to see differences in primary CD4+ cell with different stimulations, including PHA/PMA. To date, no obvious differences were seen for DNTTIP1, MIDEAS & NAT10 in Westerns blots. It is possible that we will need more elegant tools, such as phospho-specific antibodies to tease out the mechanisms of regulation.

3. The impact of the study would be increased with an in vitro analysis of the role of these proteins in reactivating latent virus from CD4 T cells from patients on suppressive ART. The analysis of patients’ cells is the standard for high impact studies of HIV-1 latency.

In the case of small molecules in preclinical studies, we agree that it is optimal to test patient-derived cells. In the case of the MiDAC complex, there are currently no small molecules that specifically target it. We hope the publication of our findings, that uncover for the first time its role in activated HIV gene expression, will now spur efforts to develop such molecules.

Reviewer #2: 1) Experiments validating the mass spec results are well done, but the evidence for NAT10 association is underwhelming. NAT10 was only confirmed in nocodozole treated cells and appears enriched in the ATCC IP as well, not only the HIV WT (Fig 2D). This would suggest NAT10 recruitment is not dependent on MIDEAS and DNTTIP1? Could the authors please address given later conclusions that NAT10 is a key lynchpin in why the ATCC sequence is not Tat-responsive.

The concern of the reviewer is valid. A challenge with NAT10 *in vitro* is that it binds non-specifically to nucleic acids, likely due to its highly basic C-terminus. The weak signal observed in lanes 5 & 6 of Figure 2D are the same as those seen for scrambled DNA, and we have found no washing conditions that completely remove it. It is in with the wildtype promoter, particularly with mitotic extracts, that a significant enrichment is observed. We explicitly state the background levels in the figure legend in the revised version. We have quantitated by image-j the signals to present a more mathematical view to the reviewer (fold enrichment with respect to 5% input on the vertical axis).

|  | Nat10 |
| --- | --- |
| HIV WT DMSO | 2.10592 |
| HIV WT Nocodazole | 3.15287 |
| HIV ATAC DMSO | 1.28343 |
| HIV ATAC Nocodazole | 1.13547 |

2) There is current literature that DNTTIP1/MiDAC localization appears to correlate with levels of H3K27ac and H4K20ac at targeted promoters which control gene expression (<https://pubmed.ncbi.nlm.nih.gov/32297854/>). Given the authors have validated siRNAs targeting these proteins and have established ChIP protocols, we would highly recommend examining the HIV LTR for changes in these marks upon MiDAC component depletion. This would lend significant weight to a potential mechanism for positive regulation by MiDAC.

In the future, investigating potential correlations with these histone marks could be of high interest. Currently, the only available information pertains to another organism (mouse versus human) and tissue (neurons versus T cells). We are presently conducting transcriptome studies in human T cells to establish the foundation for such experiments.

3) In Figure 5, the authors indicate the cells are transfected with an HIV LTR reporter plasmid and Tat expression plasmid after siRNA depletion. How was transfection efficiency controlled and standardized between samples to ensure all samples received the same amount of DNA? What do the error bars represent? Are these independent replicates performed at different times? This should be included in the figure legend and for all other figures containing error bars.

In Figure 5 the results of three independent transfections performed in parallel. The error bars represent the standard deviation of the three transfections. As is standard for transient transfections, the amount of DNA added was controlled by spectrophotometry before but not after transfection. These values are from independent replicates performed in parallel (at the same time). We have also added a sentence in the legends of Figures 4, 5, 6 & 7 to ensure the meanings of the error bars is explicit throughout the manuscript.

a. The meaning of the % Tat transactivation in this figure is extremely confusing. Does this indicate MFI relative to % positive YFP, or MFI of Tat+siRNA/NoTat+siRNA? Why is the % transactivation for NAT10 N2 80% but YFP+ cells decreased by more than 50%? Does this indicate higher MFI but in fewer cells, suggesting greater activation in a select number of cells?

We have prepared a revised version of the Figure where Tat transactivation is presented in a separate panel for clarity. To visualize siRNA-dependent changes in cells not expressing Tat (i.e., cells with very low fluorescence), the percentage of YFP-positive cells is depicted in Panel B. For direct comparison, Panel C shows the percentage of YFP-positive cells. The Tat transactivation value represented in Panel D is based on the mean fluorescence intensity (MFI) from Tat-expressing cells divided by the MFI from empty vector-expressing cells. We chose this representation because these values correlate best with those of our luciferase-based assays in the paper. N2 siRNA shows a weaker effect on Tat transactivation, whether calculated using the percentage of positive cells or mean fluorescence intensity. Overall, the impacts of the different siRNAs and shRNAs used in the manuscript have comparable effects when assayed by % YFP, MFI, or luciferase assays.

4) Figure 6C – How was the number of integrated viruses per cell determined? This was not detailed in the methods. The methods as written suggest an infection at an MOI of 1 yet this figure indicates an average of 5 viruses/cell? How is this possible given the MOI?

The number of integrated proviruses was calculated by quantitative PCR of extracted genomic DNA using HIV-specific primers (PMID: 22834489). We have clarified this in the Materials and Methods section. We estimate MOIs of viral stocks by the same technique, and of aim for one copy per cell, but the actual experimental result is virtually never exactly one. The experimental design is such that the gene depletion could conceivably have had effects on viral steps before transcription. Controlling for proviral number was important to show that is not the case. Our experience shows that the depletion results are robust with proviral copy numbers ranging from 0.2 to 8 per cell.

5) Figure 7 – The authors cite work by the Verdin and Sadowski labs performed using dual-fluorescent reporters to justify that latency is established in 48hrs in single round infections in Jurkats. While the papers cited do show rapid silencing of a population, it is not the entire population and the specific latent population can be identified and sorted via the dual-reporter system to specifically examine the early latent pool. Using a luciferase-based virus, this population cannot be sorted and it is impossible to tell if the increased luciferase is due to increased transcription from those viruses that are not immediately silenced verses from those that are. Linking these sequences to reactivation from latency is a stretch.

It is true that 10 – 30% of the cells in this system can have active transcription. Since latency is, by definition, transcriptional silence, the active population must contribute to a dampening effect on the induction of gene expression by LRAs. Despite the “leakiness” of the system, we readily observe reproducible and specific impacts of the mutations in the CTGC motifs on the induction by LRAs, strengthening the conclusion that these motifs play a major role the response to specific LRAs.

Based on the comments, we have removed the term latency from all the results and refer simply to the activation of HIV gene expression.

a. The involvement of MiDAC in latency and reactivation to various LRAs would be better reinforced with DNTTIP1/MIDEAS knockdowns in established latency models (Verdin JLat lines, Karn cell lines), followed by treatment with various LRAs to examine viral reactivation in the absence of these proteins.

Unfortunately, to date, we have not been able to achieve convincing gene knockdowns with our available tools in these cell models.

6) Overall, we are unclear as to the final mechanism being proposed. Is the primary function of MiDAC Tat-recruitment via scaffolding of NAT10? Does MiDAC induce chromatin changes at the LTR? Does MiDAC deacetylate protein targets? The ATCC mutated TASHET sequence shows significant differences in binding of proteins via EMSAs in Supplementary Figure 1 and in the Whilhem et al Retrovirology 2012 paper. While the ATAC sequence does appear more like wild-type with respect to protein binding, there does not appear to have been a truly unbiased approach to characterized all proteins bound to these oligos and there may be other changes to recruited complexes which impact overall Tat recruitment upon mutation of this core sequence. We agree that there does appear to be reduced Tat responsiveness when MiDAC components are specifically knocked down, but there is no definitive experiment that points to the mechanism of this action. Further, as everything is done in the context of a 48hr single round infection, this would suggest MiDAC is involved in aiding productive transcription in early viral infection but the relevance reactivation after long-term latency is not clear to us. Experiments suggested here, primarily ChIP for histone marks and experiments in latency lines would augment this study significantly.

These are a series of very interesting questions for which we can propose some answers, but the precise mechanism by which MiDAC controls gene expression at any promoter is not yet known. Concerning the proteomic data, we agree that it was not unbiased. Indeed, everything was designed to ensure we selected physiologically relevant proteins that bind to the wild-type sequence and not to inactive mutated sequences. We agree that looking at the proteins that bind to other mutations will be of interest, ideally using quantitative, rather than selective techniques. However, we do not yet know with confidence if these proteins represent blocked or stalled transcription complexes that have physiological impacts, or simply aberrant complexes imposed by unnatural mutations. Given that MiDAC is an HDAC complex, it would seem highly likely that one of its roles is in modifying chromatin at the HIV LTR. Since there are numerous other factors at the HIV LTR that recruit HDAC1 and HDAC2, dissecting out the specific modifications directly controlled by MiDAC will be challenging. We plan to collaborate with laboratories with specific expertise in the analysis of chromatin modifications to examine these issues in detail in the future.

Reviewer #3: Additional specific comments:

1. The complex was initially identified by purification from HeLa cells. They demonstrated a similar complex in Jurkat cells and normal T cells. Although difficult, it would be important to demonstrate effects of these proteins for expression of HIV-1 in primary cells.

As we mentioned in a previous point, we have put a significant amount of time and energy into trying to knockdown these genes in primary T cells either by lentiviral delivery of shRNA or nucleofection of siRNA without success. If any of the reviewers, or any member of the research community, is willing to share with us a robust protocol for knockdown in primary T cells we will implement it immediately. Until that time, the cell line work is what we can share.

While we agree primary cell data would be valuable, it is also worth considering the context. Of the hundreds of transcription factors thought to control HIV gene expression, including the key players like NF-kappaB, SP1 and TBP, because of the technical constraints, none has ever been shown genetically to control HIV expression in primary T cells.

2. In Figure 2D they show that the complex from cells arrested in M phase has higher affinity than cells from an unsynchronized population. Further to the comment above, what effects do arresting in G1 or S phase have for binding ability in this assay?

We have investigated this interesting question and have not seen significance difference with extracts from G1/S cells. Our observation is in line with previous observations showing the catalytic activity of the complex being specifically altered in G2/M but not G1/S (PMID: 21258344).

3. Figure 4, the ChIP experiment was performed with HeLa cells. Do they see similar interaction with the LTR in T cells?

A reasonable question. We have invested considerable efforts into the ChIP experiments in T cells. The ectopic expression of DNTTIP1 leads to cell death in all cells, but T cells are particularly sensitive to this phenomenon (see Figure below). The lethal effect of DNTTIP1 in T cells is biologically interesting, but unfortunately precludes using them for ChIPs until better antibodies are developed against endogenous DNTTIP1. HeLa cells also presented challenges for ChIPs experiments since they also die, albeit at a manageable rate (see below).

4. Figure 4, the DNTTIP1 ChIP also shows significant interaction in the upstream LTR region. Is there a TASHET-like sequence in this region that could account for this?

The reviewer makes an astute observation. We have previously noted CTGC motifs upstream of the NF-kappaB sites in the LTR (PMID: 9000143) that could potentially be recognized by DNTTIP1. However, the resolution of the ChIP assay does not allow us to make such a claim for the time being.

5. In Figure 5 with the siRNA experiments, there seems to be a significantly different effect of the two different DNTTIP1 siRNAs, yet these cells have comparable levels of protein knockdown. Does expression of TAT affect the efficiency of knockdown in these experiments?

No, across experiments, Tat does not affect the knockdown. We have used at least two independent shRNA in multiple cell lines and independent siRNA in multiple cells lines with consistent results and with different experimentalists to be confident of the knockdowns. For these knockdown targets and other positive controls like TBP, we do not always observe a perfect correlation between protein levels and the impact on gene expression.

6. In Figure 6C, this analysis seems to involve cells that have ~5 provirus per cell. It would be important to determine the effect of knockdown on cells containing a single provirus integration, as would be typical for a normal HIV-1 infection.

As we mentioned above, the depletion experiments give robust impacts on HIV gene expression in cells with proviral copy numbers ranging from 0.2 to 8 per cell. We have not tried less or more since we aim for approximately one copy per cell, but rarely achieve exactly one due to variability in infection rates. The reason that we measure and report the copy number is to ensure is does not vary due to knockdown effects on steps earlier in the viral lifecycle than transcription.

**Part III – Minor Issues: Editorial and Data Presentation Modifications**
Please use this section for editorial suggestions as well as relatively minor modifications of existing data that would enhance clarity.

Reviewer #1: 1. Lines 95-96 seem to state that Tat binds to TAR RNA and then recruits P-TEFb. It has been established first by Katherine Jones that Tat and Cyclin T bind TAR RNA together and likely with CDK9. See Jones’ Cell paper in 1998 and a number of subsequent papers from numerous laboratories.

The text has been revised not to imply an order of binding of Tat & Cyclin T1 (lines -). Thank you.

2. Why was NAT10 not evaluated in Figure 2A-C?

DNTTIP1 and MIDEAS were evaluated in early experiments because they were known to form a tight complex. NAT10 was not known to be part of such a complex. We began to assess NAT10 later in the project, given the fact that two independent groups reported its interaction with HIV Tat. Unfortunately, a single blot cannot be used for the MIDEAS and NAT10 because of their similar mobilities on SDS-PAGE gels.

3. Line 198. Authors state that “Overall, our results reveal that MiDAC complex subunits MIDEAS, DNTTIP1 and HDAC1 selectively interact with the wild type TASHET element of the HIV-1 core promoter and that they also recruit NAT10 to TASHET.” No evidence is presented that these proteins recruit NAT10.

We have removed the statement about NAT10 recruitment in the results section as indicated (lines 203 - 205).

Reviewer #2: 1) Please clarify in the methods if corresponding gel areas were extracted for mass spec in Fig 1 in the ATCC/ATAC enrichments or only in the WT sample for mass spec.

The corresponding gel area was not for MS because there were no visible corresponding bands to excise. We have clarified this in the Materials and Methods section (lines 701 - 703).

2) Redundant sentences page 17, lines 378-381.

Thank you, the redundant sentence has been removed (lines 387 - 390).

3) How is Figure 7 standardized? Is it to each individual virus DMSO control or all to WT virus?

Figure 7 is normalized to each individual virus and we have clarified the point by the adjusting the text of the figure legend (lines 1423 - 1424).

Reviewer #3: (No Response)

We thank in you kindly for your time and consideration,

Brendan Bell, PhD
